# Assessment of oral health-related quality of life after total temporomandibular joint replacement

**DOI:** 10.22514/jofph.2025.032

**Published:** 2025-06-12

**Authors:** Alexandre Weber, Guilherme Ommizolo, Vanessa Rente, Roberto Ferreira Zanin, Cláiton Heitz, Eduardo Martinelli de Lima

**Affiliations:** ^1^Department of Stomatology, Federal University of Santa Maria, 97105-900 Santa Maria, RS, Brazil; ^2^Oral and Maxillofacial, Hospital São Miguel, 89600-000 Joaçaba, SC, Brazil; ^3^School of Dentistry, School of Health Sciences, Federal University Santa Maria, 97105-900 Santa Maria, RS, Brazil; ^4^Oral and Maxillofacial Surgery, Post-graduate Program in Dentistry, School of Health Sciences, Pontifical Catholic University of Rio Grande do Sul, 90619-900 Porto Alegre, RS, Brazil; ^5^Orthodontics, Post-graduate Program in Dentistry, School of Health Sciences, Pontifical Catholic University of Rio Grande do Sul, 90619-900 Porto Alegre, RS, Brazil

**Keywords:** Temporomandibular joint replacement, Oral health-related quality of life, Advanced temporomandibular joint disorder, OHIP-14 questionnaire

## Abstract

**Background**: The article addresses the impact of total temporomandibular joint replacement (TMJR) on oral health-related quality of life (OHRQoL) in patients with advanced temporomandibular joint (TMJ) disorder. TMJ disorder encompasses a variety of conditions causing pain and dysfunction in the jaw, peripheral nerves, and TMJ. TMJR is considered a last resort in the surgical treatment of advanced joint disorders when conservative treatments fail. **Methods**: The study followed 10 patients undergoing TMJR and assessed their oral health-related quality of life using the OHIP-14 (Oral Health Impact Profile-14) questionnaire at different postoperative time points. **Results**: The results showed a significant improvement in physical pain, psychological discomfort, and physical disability after six months, with a restoration of oral health-related quality of life after twelve months. **Conclusions**: The research concluded that TMJR treatment can significantly improve oral health-related quality of life in patients with advanced TMJ disorder. However, further studies are needed to analyze the long-term effectiveness of treatment and compare different surgical approaches.

## 1. Introduction

The temporomandibular joint (TMJ) is a sophisticated joint essential for human movement and function. It serves as the connection point between the mandible and the temporal bone of the skull, facilitating various movements of the jaw. Comprising two condyles located at either end of the mandible, the TMJ operates seamlessly. Unlike typical joints, the articulating surfaces of the bones do not directly contact each other. Instead, they are separated by a specialized disc, which acts as a cushion, absorbing stress and enabling smooth movement of the condyles during the opening and closing of the oral cavity. This disc effectively divides the TMJ into two synovial cavities, each lined with synovial membranes. Furthermore, the articulating surfaces of the bones are coated with fibrocartilage, providing stability and support distinct from the more common hyaline cartilage found in other joints [1].

Total temporomandibular joint replacement (TMJR) is generally considered the last resort in the surgical treatment of end-stage TMJ disorders, especially when conservative treatments have failed. The TMJ is a complex joint, essential for chewing, speaking, and other daily functions, with a unique anatomy that includes a specialized disc that separates the articular surfaces of the bones. Understanding the detailed anatomy of the TMJ is crucial for effective surgical planning and execution of the procedure [2, 3].

Temporomandibular disorders (TMDs) are a subcategory of orofacial pain conditions, which encompass pain in the hard and soft structures of the head, face and oral cavity. TMJ disorders specifically involve dysfunctions in the TMJ and associated structures, often leading to significant pain and functional limitations. Orofacial pain, in a broader sense, includes conditions such as dental pain and neuropathic pain, but TMJ disorder are distinguished by their direct impact on the TMJ and related musculature [1, 4].

The TMJR procedure has been shown to improve the emotional state of patients with end-stage TMJ disorder [3]. TMDs, including TMJ disorders, have a significant impact on oral health-related quality of life (OHRQoL) [5, 6]. Patients with TMDs experience symptoms such as chronic pain, restriction of function and psychological effects, which negatively affect their overall quality of life [7]. The management of TMDs should consider the impact of the condition on the daily life of individuals and incorporate interventions that address both the physical and psychological aspects of the condition [8]. Studies show that anxiety, often associated with TMDs, can exacerbate the perception of pain and functional limitation, contributing to a decline in patient quality of life [9]. It is essential to consider the mental burden of patients during the treatment and management of TMDs, since psychological factors, such as anxiety and stress, can significantly influence treatment outcomes. Therefore, when considering treatment and maintenance for TMD patients, both their physical conditions and anxiety levels should be taken into account. TMJR offers patients the opportunity for improved function and reduced pain, which can positively impact their emotional state.

The evolution of TMJR techniques, as detailed by Mercuri LG *et al*. [10] (2022), reflects significant progress in surgical approaches and materials used. Additionally, the review by Lima FGGP *et al*. [11] (2023) provides a comprehensive overview of the long-term survival of prostheses, reinforcing the efficacy of the procedure in patients with severe TMJ disorders.

Performing TMJR in patients with end-stage TMJ disorder carries certain risks. The most prevalent complications include paresis or paralysis of the facial nerve branches, sensory alterations, heterotopic bone formation and infection [3]. Additionally, a study found that 20.5% of patients experienced chronic postoperative pain after TMJ replacement surgery, with severe preoperative pain scores, regular opioid use, and multiple previous open TMJ surgeries being predictive risk factors for persistent pain [4, 12, 13]. However, TMJR is considered a standard procedure for end-stage TMJ disorder, providing restoration of form and function, improvement in quality of life, reduction in pain, and maintenance of ramal height [1]. It is important for maxillofacial surgeons to be well acquainted with TMJR [14]. The surgery demands careful patient selection and ongoing development of appropriate clinical guidelines [8]. Psychological factors such as anxiety have also been observed in TMD patients, but there is no significant difference in the incidence of depression and somatic symptoms compared to normal prosthodontics outpatients [8, 9, 15].

The etiology of TMDs is multifactorial, including trauma, genetic and hormonal factors, as well as systemic conditions such as rheumatoid arthritis. Epidemiological studies indicate that the prevalence of TMDs is higher in women, particularly those between the ages of 20 and 40, suggesting a possible hormonal influence. The relationship between the etiology of TMDs and their prevalence in the female population highlights the need for a differentiated approach in the treatment and management of these conditions [9].

The aim of this study was to evaluate the impact of TMJR on OHRQoL in patients with end-stage TMJ disorder using the OHIP-14 questionnaire.

## 2. Methods

The study design is a prospective case series without a control group. Therefore, the results should be interpreted in the context of epidemiological or normative data. The research was approved by the Institutional Research Ethics Committee (approval number: 70056217.4.0000.5336/2017).

The definition of “end-stage TMJ disorder” in this study refers to conditions with severe joint dysfunction, chronic pain unresponsive to conservative treatment, and significant TMJ degeneration. The pathologies included ankylosis, severe condylar resorption, and advanced osteoarthritis. Diagnosis was based on clinical evaluation, medical history, Cone Beam Computed Tomography (CBCT), Magnetic Resonance Imaging (MRI) and confirmation by an oral and maxillofacial surgeon, ensuring a rigorous classification of end-stage TMJ disorder.

The eligibility criteria included patients with severe TMJ disorder treated with TMJR between September 2017 and September 2023. All individuals were invited to participate in the study, received information about the study objectives, and signed an informed consent form before enrollment.

The criteria for indicating TMJR followed guidelines from the American Association of Oral and Maxillofacial Surgeons (AAOMS). TMJR was indicated for patients with severe chronic pain, significant joint dysfunction and irreversible joint degeneration unresponsive to conservative treatments. Treatment failure was defined after at least six months of continuous conservative therapy, including medication, physical therapy, occlusal therapy and intra-articular injections, without significant symptom relief or functional improvement.

In the study, the null hypothesis (H_0_) proposes that there is no significant difference in OHIP-14 scores before and after TMJR across the evaluation periods (two months, six months and twelve months). Conversely, the alternative hypothesis (H_1_) suggests that there is a significant difference in OHIP-14 scores over these periods, indicating a notable impact of TMJR on the OHRQoL of patients.

### 2.1 Inclusion and exclusion criteria

The study included healthy individuals over 18 years of age with end-stage TMJ disorder, a history of unsuccessful conservative treatment, and an indication for unilateral or bilateral TMJR. Patients with postoperative trauma, those lost to follow-up, or those unable to fill out the OHIP-14 form were excluded.

### 2.2 Sample

Ten patients (four men, six women; mean age 37 ± 10 years) met the inclusion criteria and were included in the study. In addition to demographic information, detailed measurements of mandibular range of motion were collected, including maximum mouth opening, protrusion, and lateral movements at different postoperative time points. Pain intensity was also assessed using visual analog scales (VAS), providing a comprehensive overview of patient status over time (Table 1).

**Table 1.** Description of the sample (N = 10).

Id	Sex	Age (yr)	Previous surgery	Etiology	Side		Prosthesis type
# 1	F	55	No	Trauma (ankylosis)	Left	Right	Standard
# 2	M	50	No	Trauma (ankylosis)	Left	Right	Standard
# 3	F	46	No	Ankylosing spondylitis	Left	Right	Customized
# 4	F	42	No	Idiopathic condylar atrophy	Left	Right	Customized
# 5	M	18	No	Trauma (ankylosis)	Left	-	Customized
# 6	F	31	No	Idiopathic condylar atrophy	-	Right	Customized
# 7	M	37	Fracture	Trauma (ankylosis)	-	Right	Standard
# 8	F	34	Discopexy	Rheumatoid arthritis	Left	Right	Standard
# 9	F	29	No	Idiopathic condylar atrophy	-	Right	Customized
# 10	M	31	No	Trauma (ankylosis)	-	Right	Standard

*Id means patient identification; yr: years; M: male; F: female.*

### 2.3 Surgical procedures and postoperative strategy

Before surgery, the patients used non-steroidal anti-inflammatory drugs (NSAIDs), the glucocorticoid dexamethasone (4 mg), and a 0.12% chlorhexidine solution mouthwash for three days. TMJR was performed in the surgical ward under general anesthesia with nasal intubation. Asepsis included the use of 2% chlorhexidine solution in the surgical field and 0.12% intraorally. Pre-auricular incisions were made, and access to the joint was obtained using appropriate approaches to avoid damage to facial nerves.

Postoperative management followed the protocol of the Hospital, including intravenous morphine, antibiotics, NSAID analgesics and muscle relaxants, along with ice therapy in the first 48 h.

### 2.4 Rehabilitation and physiotherapy

Postoperatively, all patients were referred to physiotherapy, where they performed specific exercises to restore mandibular range of motion. They were instructed to perform passive and active mouth opening exercises, as well as lateral and protrusive movements, under the supervision of a physiotherapist specialized in TMDs. This rehabilitation was crucial to maximize functional recovery and minimize joint stiffness. Patients were periodically monitored to assess adherence to the exercise regimen and progress in restoring mandibular function.

Physiotherapy rehabilitation is initially based on facial drainage in the event of edema, muscle relaxation, and gaining TMJ mobility through myofascial release techniques for the masseter, temporalis, medial pterygoid and lateral pterygoid muscles, together with the release of the infrahyoid and suprahyoid muscles. After the structures are relaxed, exercises are carried out for joint gain.

The exercises are based on:

• Opening and closing the mouth;

• TMJ stretching: where the patient places the tip of the tongue on the roof of the mouth without touching the teeth at the top, applying gentle pressure when opening and closing the mouth;

• Cheek movement: the patient inflates their cheeks with air and moves the air from one side of their cheeks to the other with their lips closed. This helps to relax the masticatory muscles, facial muscles, and facial bone structures;

• Anteriorization of the mandible: the patient moves the mandible with an antero-posterior movement;

• Lateralization of the mandible: the patient moves the mandible to the right and left sides;

• All exercises must be carried out respecting the range of joint movement and pain threshold of each patient.

Other muscular structures can also be assessed by the professional, such as the cervical and cranial regions, for possible secondary tensions due to the surgical procedure.

### 2.5 Data analysis

Mandibular range of motion and pain scores were recorded preoperatively (T0) and at follow-up intervals of two months (T1), six months (T2) and twelve months (T3). Statistical analysis was performed using the Friedman non-parametric test to compare OHIP-14 scores, mandibular range of motion, and pain intensity between different time points. The R program (version 3.3.1, R Foundation for Statistical Computing, Vienna, W, Austria) was used for analysis, with the significance level set at 5%. The means and standard deviations for VAS scores at each assessment time point are presented in Table 2.

**Table 2.** Means and standard deviations for visual analogue scale (VAS) scores at each assessment time point.

Symptom	T0	T1	T2	T3
TMJ pain	7.04 (1.85)	5.71 (3.24)	2.53 (2.01)	0.44 (0.65)

*T0: preoperatively; T1: follow-up intervals of two months; T2: follow-up intervals of six months; T3: follow-up intervals of twelve months; TMJ: temporomandibular joint.*

## 3. Results

Before TMJR, mean scores in domains physical pain (3.4), physical disability (2.2) and functional limitation (1.1) accounted for 55% of the total OHIP-14 score (12.3); psychological discomfort (2.9) and psychological disability (1.7) accounted for 38%; and social disability (0.6) and handicap (0.3), only 7%; meaning that TMJ disorder was mainly a physical problem. In the two-month follow-up (T1), all domain scores decreased with no statistical significance (*p* > 0.05). Six-month follow-up (T2) showed statistically significant score decreases in physical pain, psychological discomfort, physical disability and psychological disability, as well as in total OHIP-14 score (*p* < 0.001). Functional limitation improved significantly at twelve-month follow-up (T3) (*p* < 0.001). Social disability and handicap showed no statistically significant score changes throughout (*p* > 0.05). No domains had significantly score variation between subsequent evaluations (*p* > 0.05) (Table 3).

**Table 3.** Descriptive statistics of OHIP-14 scores by domain (N = 10).

	Baseline		Two-month		Six-month	Twelve-month		
Domain	T0 Score Mean ± SD	T1 Score Mean ± SD	T1−T0 % Mean	T2 Score Mean ± SD	T2−T0 % Mean	T3 Score Mean ± SD	T3−T0 % Mean	*p*
Functional limitation	1.1 ± 1.0	0.5 ± 0.6	−54	0.2 ± 0.3	−82	0.1 ± 0.2	−90*	<0.001
Physical pain	3.4 ± 0.6	1.5 ± 0.5	−56	0.5 ± 0.4	−85*	0.1 ± 0.2	−97*	<0.001
Psychological discomfort	2.9 ± 0.9	1.3 ± 0.9	−55	0.8 ± 0.6	−72*	0.1 ± 0.2	−97*	<0.001
Physical disability	2.2 ± 1.5	0.9 ± 0.9	−59	0.4 ± 0.5	−82*	0.1 ± 0.3	−95*	<0.001
Psychological disability	1.7 ± 0.9	0.6 ± 0.7	−65	0.2 ± 0.3	−88*	0	−100*	<0.001
Social disability	0.6 ± 0.8	0.4 ± 0.5	−33	0.2 ± 0.3	−67	0.1 ± 0.2	−83*	0.120
Handicap	0.3 ± 0.7	0.2 ± 0.5	−33	0.1 ± 0.2	−67	0	100	0.110
Total OHIP-14	12.3 ±5.2	5.7 ± 3.2	−54	2.5 ± 2.0	−80*	0.4 ± 0.6	−97*	<0.001

Friedman test (p < 0.05). *: statistical significance; SD: standard deviation; OHIP-14: Oral Health Impact Profile-14.

The domains of functional limitation, physical pain, psychological discomfort, physical disability, psychological disability, social disability and handicap were analyzed. These aspects were thoroughly examined in separate figures presented below. In addition, details of the figures below can be found in the Methods section (Tables 4 and 5).

Similar to the study by Rauniyar D *et al*. [16] (2023), our findings indicate that TMJR can provide significant improvement in joint function for patients with recurrent ankylosis.

**Table 4.** Description of OHIP-14 domain items.

Domain	Item	Question
Functional Limitation	1	Have you had trouble pronouncing a word?
	2	Have you felt that the taste of food has gotten worse?
Physical Pain	3	Have you felt severe pain in your mouth?
	4	Have you felt uncomfortable eating any food?
Psychological Discomfort	5	Have you have been feeling uncomfortable?
	6	Have you felt stressed?
Physical Disability	7	Has your diet been compromised?
	8	Have you had to interrupt your meals?
Psychological Disability	9	Have you found it difficult to relax?
	10	Have you felt a little embarrassed?
Social Disability	11	Have you been irritable with other people?
	12	Have you had difficulty carrying out your daily activities?
Handicap	13	Have you felt that life in general has gotten worse?
	14	Have you been unable to do your daily activities?

**Table 5.** Description of OHIP-14 test scores.

Score	Answer
0	Never
1	Rarely
2	Sometimes
3	Fairly often
4	Very often

## 4. Discussion

The results of this study demonstrated a significant improvement in OHRQoL following TMJR in patients with advanced TMJ disorder. These findings are consistent with previous studies that have also reported substantial improvements in OHRQoL after TMJR. For instance, Torres KV *et al*. [17] (2017) found that patients undergoing orthognathic surgery, which may also involve the TMJ, experienced significant enhancements in quality of life, comparable to those observed in our study.

At baseline (T0), physical pain (item 3) and psychological discomfort (item 5) received score 3 (fairly often) or 4 (very often) from all patients, meaning a higher OHRQoL impact; whereas social disability (item 11) and handicap (item 14) received score 0 (never) or 1 (rarely), meaning lower OHRQoL impact. Two month-follow-up (T1) showed no score 4 in any domain and 10% score 3 in functional limitation, psychological discomfort and physical disability. In the six-month follow-up (T2), no domain received score 3 or 4. At twelve months after TMJR (T3), all domain scores were 0 or 1, suggesting OHRQoL improvement in all patients (Figs. 1,2,3,4,5,6).

**Fig. 1.** Distribution of domain scores by evaluation (N = 10). T0: preoperatively; T1: follow-up intervals of two months; T2: follow-up intervals of six months; T3: follow-up intervals of twelve months.

**Fig. 2.** Distribution of domain physical pain. T0: preoperatively; T1: follow-up intervals of two months; T2: follow-up intervals of six months; T3: follow-up intervals of twelve months.

**Fig. 3.** Distribution of domain psychological discomfort. T0: preoperatively; T1: follow-up intervals of two months; T2: follow-up intervals of six months; T3: follow-up intervals of twelve months.

**Fig. 4.** Distribution of domain physical disability. T0: preoperatively; T1: follow-up intervals of two months; T2: follow-up intervals of six months; T3: follow-up intervals of twelve months.

**Fig. 5.** Distribution of domain social disability. T0: preoperatively; T1: follow-up intervals of two months; T2: follow-up intervals of six months; T3: follow-up intervals of twelve months.

**Fig. 6.** Distribution of domain handicap. T0: preoperatively; T1: follow-up intervals of two months; T2: follow-up intervals of six months; T3: follow-up intervals of twelve months.

The findings of this study are consistent with the systematic review conducted by Neuprez A *et al*. [18] (2020), which also reported substantial improvements in quality of life following TMJR.

The results of the present study are also consistent with the conclusions of Jones & Roberts (2022), who observed a significant improvement in patient quality of life following TMJR in a longitudinal study. Additionally, the meta-analysis conducted by Neuprez A *et al*. [18] (2020), corroborates these findings, highlighting the positive impact of the procedure on OHRQoL [19].

When comparing our results with those of prosthetic replacements of other joints, such as hip and knee, we find both similarities and differences. Studies on hip and knee arthroplasties have demonstrated that joint replacements also result in significant improvements in patient quality of life, particularly in terms of pain reduction and increased mobility [18, 20, 21, 22]. However, the TMJ is a complex joint that plays a crucial role in mastication, speech, and other daily functions, making the functional demands postoperatively different from those of other joints.

A distinctive aspect of our study is the use of the OHIP-14 questionnaire to evaluate OHRQoL, allowing for a detailed analysis of changes in specific domains such as physical pain, psychological discomfort, and functional limitation. In contrast, studies on hip and knee arthroplasties often use tools such as the Western Ontario and McMaster Universities Arthritis Index (WOMAC) or the Hip Disability and Osteoarthritis Outcome Score (HOOS), which, while effective for those joints, do not capture the specific nuances of mandibular function [23].

Furthermore, it is important to note that, similar to other joint arthroplasties, postoperative rehabilitation plays a critical role in patient recovery after TMJR. The use of targeted physiotherapy, as conducted in our study, has proven essential to maximize functional recovery and minimize joint stiffness, akin to practices observed in hip and knee rehabilitation.

However, unlike larger joints where functional recovery can be more easily monitored and compared with established benchmarks, the assessment of TMJ function requires a more individualized approach, given the variability in patient responses to surgery and rehabilitation.

In addition, a study by Zanin RF & Weber A *et al*. [24] (2019) also revealed significant improvements in OHRQoL after surgical interventions in the TMJ, corroborating the findings of the present research. On the other hand, a study by De Laura TL *et al*. [25] (2023) further emphasizes the importance of TMJR in improving OHRQoL, showing that TMJR, as other arthroplasties, has a positive and lasting impact on patient quality of life.

In conclusion, the results of this study underscore the efficacy of TMJR in improving the quality of life of patients with advanced TMJ disorder, with outcomes that are, in many respects, comparable to those observed in other joint replacements. However, the functional complexity and importance of the TMJ in daily life require a carefully planned and personalized approach to treatment and rehabilitation.

The study faced limitations due to the small sample size and the diversity of TMJ disorder etiology among patients. However, TMJR is still not a common treatment option in Brazil, where the study was conducted, and conservative methods such as NSAIDs, physical therapy, and less invasive surgical approaches are preferred. Nonetheless, evidence-based studies have demonstrated significant benefits of TMJR in both the short and long term. Future studies should analyze the differences in OHRQoL impact between TMJR with standardized and customized devices, as well as compared to conservative surgical approaches [20, 21].

## 5. Conclusions

Under the limitations of the study and according to the methodology followed, it is possible to conclude that treatment of end-stage TMJ disorder with TMJR caused significant improvement in patient perception of OHRQoL at six months after surgery, whereas OHRQoL was reestablished after twelve months.

The present study underscores the guidelines by Monje Gil, Florencio *et al*. [26] (2024) on the efficacy of TMJR in improving patient quality of life.
